# Unveiling the black box: A systematic review of Explainable Artificial Intelligence in medical image analysis

**DOI:** 10.1016/j.csbj.2024.08.005

**Published:** 2024-08-12

**Authors:** Dost Muhammad, Malika Bendechache

**Affiliations:** ADAPT Research Centre, School of Computer Science, University of Galway, Galway, Ireland

**Keywords:** Explainable AI, Medical image analysis, XAI in medical imaging, XAI in healthcare

## Abstract

This systematic literature review examines state-of-the-art Explainable Artificial Intelligence (XAI) methods applied to medical image analysis, discussing current challenges and future research directions, and exploring evaluation metrics used to assess XAI approaches. With the growing efficiency of Machine Learning (ML) and Deep Learning (DL) in medical applications, there's a critical need for adoption in healthcare. However, their “black-box” nature, where decisions are made without clear explanations, hinders acceptance in clinical settings where decisions have significant medicolegal consequences. Our review highlights the advanced XAI methods, identifying how they address the need for transparency and trust in ML/DL decisions. We also outline the challenges faced by these methods and propose future research directions to improve XAI in healthcare.

This paper aims to bridge the gap between cutting-edge computational techniques and their practical application in healthcare, nurturing a more transparent, trustworthy, and effective use of AI in medical settings. The insights guide both research and industry, promoting innovation and standardisation in XAI implementation in healthcare.

## Graphical abstract

**Figure:**
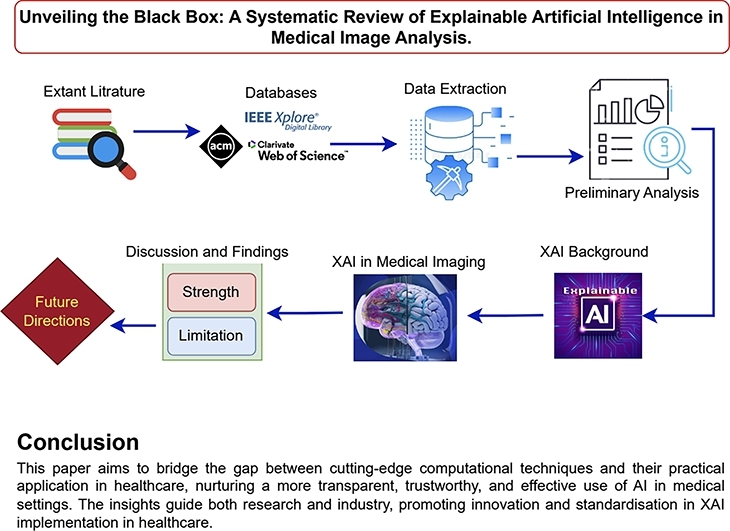

## 1 Introduction

Over the last ten years, the employment of artificial intelligence (AI) driven by machine learning (ML) and deep learning (DL) has shown impressive effectiveness in the medical field for various tasks, such as diagnosis of brain and breast cancer [1], [2], detection of retinal disease [3] and medical image segmentation [4]. Notwithstanding these advances, the integration of deep neural networks (DNN) into various clinical practice contexts has been sluggish and has not gained widespread acceptance in the medical community. This hesitancy is mostly caused by the propensity to score the model performance over the explainability of decision-making procedures [5]. Explainability is a valuable tool that can be used to evaluate and improve performance by pinpointing areas of weakness, recognising hidden patterns within the input data, and identifying clinically irrelevant features among many input parameters and network layers [6]. Most eminently, utilising Explainable AI (XAI) enhances clinicians' trust in their decision-making processes by improving the transparency of healthcare algorithms.

According to the Defence Advanced Research Projects Agency (DARPA) [7], XAI endeavours to generate models that are increasingly interpretable and explainable while upholding a superior level of learning efficacy (prediction performance), empowering human users to comprehend, place appropriate trust in, and effectively manage the emerging generation of artificially intelligent partners. Despite its broad applicability, XAI holds particular significance in critical decisions, notably in clinical practice, where erroneous judgements could have grave consequences, potentially resulting in the loss of human life. This is further supported by the European Union's General Data Protection Regulation (GDPR), which mandates transparency in algorithmic decision-making processes before their employment in patient-care settings [8]. Additionally, according to the U.S. Department of Health and Human Service's final guidance on Clinical Decision Support Software (CDSS), understanding regulatory requirements is crucial to ensure these systems are safe and effective for clinical use. XAI enhances this by providing explainability features that improve transparency and reliability, helping meet regulatory standards and supporting informed clinical decisions.^1^

### 1.1 Comparison with established works

Acknowledging the importance of explainability and its pivotal role in producing reliable and trustworthy AI, researchers have embarked on comprehensive reviews of the extant XAI techniques. The comprehensive explanations covering general XAI concepts, taxonomy, diverse definitions, evaluation of complex models, programming implementations, research topics concerning explainability, challenges encountered, and guidelines for responsible AI have been recorded in [9], [10], [11], [12], [13]. The authors of [14] conducted a systematic literature review from 2012 to 2021 in PubMed, EMBASE, and Compendex databases. They proposed the INTRPRT guidelines for human-centered design, focusing on design principles and user evaluations. However, the review is technically lacking a comprehensive discussion on XAI methods. Furthermore, the authors in [15], [16] reviewed recent advances in explainable deep learning applied to medical imaging, focusing on post hoc approaches. Moreover, comparative analyses between post hoc and intrinsic model explanations for convolutional neural networks (CNN) were conducted in [17], also presented the XAI taxonomy and recommended the future research directions. A systematic literature review of the role of XAI in combating the pandemic presented by researchers [18] investigated XAI applications in data augmentation, outcome prediction, unsupervised clustering, and image segmentation. Further, the authors of [19], [20] proposed using XAI to classify deep learning-based image analysis methods and surveyed XAI papers up to October 2022. However, they did not explain the technical workings or mathematical foundations of these methods and only reviewed a few specific techniques. Additionally, the authors of [21] categorised XAI approaches as saliency-based, while in [22], the discussion was extended to methodologies beyond saliency-based in their review papers. The mentioned studies have covered general XAI concepts, taxonomies, definitions, and the application of explainable deep learning in medical imaging, particularly focusing on post hoc approaches. However, these reviews often lack a detailed investigation of the specific evaluation criteria, disease contexts, and data relevant to medical imaging. Additionally, comparative analyses of different XAI approaches—including their mathematical foundations, working procedures, strengths, weaknesses, challenges, and practical recommendations—remain under-explored in this context.

### 1.2 Aims of this review

In contrast to the extant literature, this study aims to fill a critical research gap by offering a thorough review of XAI techniques employed specifically for medical imaging applications. It not only blends various evaluation metrics, diseases, and datasets appropriate to this domain but also meticulously outlines the strengths, weaknesses, challenges, and recommendations of each XAI category. Additionally, it provides comparative analyses of different XAI approaches, including their mathematical foundations and working procedures. By focusing on enlightening future research directions, this comprehensive review contributes substantially to advancing the understanding and application of XAI methodologies in the medical imaging context. Furthermore, it provides future directions for XAI, which would be of interest to clinicians, medical researchers, patients and AI model developers.

The rest of the paper is structured as follows: Section 2 presents the foundational background and introduces a taxonomy of XAI. Section 3 elaborates on the methodological framework adopted in this study. Section 4 highlights the results of XAI methods pertinent to medical image analysis. Section 5 also sheds light on the limitations inherent to current practices and proposes prospective avenues for future research within the domain of medical image analysis.

## 2 Background

This section provides a comprehensive background on the use of XAI in medical imaging. Additionally, we define the different types of medical images, such as Fundus images, Endoscopy, X-rays, MRI, and CT scans.

The journey into the realm of explainable expert systems began in the mid-1980s [23], although the term XAI, denoting Explainable Artificial intelligence, was first introduced by [24] in 2004. XAI's prominence rose sharply with the advancement of deep learning-based models in the industry. In 2015, the Defence Advanced Research Project Agency (DARPA) launched the explainable AI program, aiming to foster the development of ML and DL models that are not only explainable but also engender greater confidence and trust among users due to their enhanced understanding and interpretability [23]. Following this, the European Union passed regulations on the “right to algorithmic explanations” providing individuals with the right to be informed about the algorithm's decision-making process utilising their data [25]. This legal move prompted a pivot in research focus towards developing models that place higher importance on being explainable rather than just accurate. Therefore, the area XAI has seen a considerable expansion in interest within the research community, with a notable uptick in related academic publications emerging in recent years. To provide a comprehensive understanding of XAI, it is essential to understand the following key terms:

• **Explainability:** This refers to the extent to which an AI model's decision-making process can be understood by humans. It involves providing clear and interpretable insights into how the model arrives at its conclusions or decision, facilitating user trust and validation of the results.
• **Interpretation:** Interpretation pertains to the ability to provide meaningful explanations for the AI model's predictions and behaviour. This involves translating the model's internal mechanisms into human-understandable terms, often through visualizations, feature importance scores, or natural language descriptions.
• **Reliability:** In the context of XAI, reliability refers to the consistency and dependability of the AI model's explanations and predictions. A reliable XAI system should produce stable and repeatable results under similar conditions, ensuring that the explanations are trustworthy and robust.
• **Robustness:** Robustness denotes the AI model's ability to maintain its performance and provide accurate explanations despite the presence of noise, perturbations, or adversarial attacks. A robust XAI system should be resilient to variations in input data and continue to offer meaningful and accurate explanations across different scenarios.

### 2.1 XAI's types for medical data

**Fig. 1:**
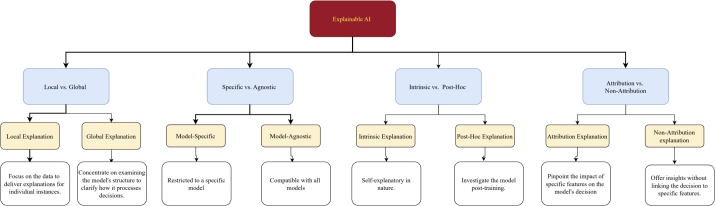
Proposed framework for categorizing XAI methods based on taxonomies in extant literature.

Explainability arises from the notion that no single algorithm stands as the ultimate solution for all types of problems better than every other algorithm. Instead, combining multiple approaches in a hybrid strategy often results in more robust solutions. Explainability methods can be categorised into the following four major categories illustrated in Fig. 1, each contributing to a deeper understanding and greater transparency of the algorithmic process.

#### 2.1.1 Local vs. global explanation

The local and global explanations serve as pivotal methodologies for demystifying the decision-making process of ML and DL models to bridge the connection between human intuition to machine logic [26]. The local explanation approach concentrates on specific data instances to interpret the rationale behind the model's decision based on the input features. This approach reveals how certain features significantly influence the model decision positively or negatively. On the other hand, the global explanation aims to understand the model's behaviour as a whole, providing a broad overview of its intelligence. For instance, identifying key features (input) that enhance the model's overall performance falls under global explanation techniques.

#### 2.1.2 Specific vs. agnostic model

In the realm of XAI, fostering trust and ensuring transparency requires a deep understanding of the ML and DL model's decision-making process through the model-specific and agnostic model [27]. The model-specific technique draws upon the distinct architecture and parameters inherent to a model, aiming to provide explanations for a particular structure. In contrast, the agnostic approach is marked by its independence from the underlying model architecture and can be deployed to other domains without directly engaging with the model's weights and parameters [28].

#### 2.1.3 Intrinsic vs. Post-Hoc explanation

Intrinsic and Post-Hoc are foundational approaches [29], marked as essential methods for demystifying the inner workings of ML and DL models. Intrinsic techniques are seamlessly integrated into the model, offering inherent interpretability with the support of different models, including decision trees and rule-based models [30], [24]. Conversely, Post-Hoc methods maintain independence from model architecture, allowing for their application across a variety of trained CNN and Vision Transformer (ViT) models without affecting the model's accuracy.

#### 2.1.4 Attribution vs. non-attribution explanation

The attribution and non-attribution methodologies are utilised as XAI tools for dissecting and understanding the predictive decision-making process of ML and DL models [31]. An attribution-based approach produces a visual explanation by illuminating specific regions of an image that are relevant to the model's prediction, achieved through a localization map. However, non-attribution approaches focus on uncovering the process and reasons that underpin a model's prediction, providing explanations that extend beyond pixel-level analysis. These methods investigate the model's working dynamics, sensitivity and stability and provide valuable insights for debugging purposes [32].

### 2.2 XAI methods based on medical imaging

XAI approaches for medical imaging are at the forefront of bridging the gap between human intuition and the complex decision-making process of ML and DL models, particularly in the realm of visual data. These techniques highlight the critical region within images that captivate the model's focus and unlock a new dimension of insight, making the complex decision easy to understand. Additionally, methods like the counterfactual technique generate comparable examples that produce different responses from the DL-models, thereby further enhancing interpretability. The utilized approaches for medical image analysis in the considered literature are listed and discussed as follows:

#### 2.2.1 Local Interpretable Model-Agnostic (LIME)

In the notion of medical image analysis, LIME is the XAI approach developed by [33] to explain the prediction of any ML or DL model in a layman-understandable manner. LIME interpret how the features or area of an input image contribute to a model's decision (prediction), by creating a local surrogate that simplifies and interprets the model's behaviour around the specific input. LIME explains by perturbing the input images, observing the changes in the model prediction and pinpointing the image features that substantially impact the model's prediction as shown in Eq. (1) [33].

(1) $$L(f,g,\pi_{x})+\Omega (g)=\sum_{i=1}^{N}w_{i}\cdot {\left(f(x_{i})-g(x_{i}^{\prime })\right)}^{2}+\Omega (g).$$

In Equation (1), *f* and *g* are functions with different inputs. Specifically, $f(x_{i})$ denotes the function *f* applied to the original input $x_{i}$, while $g(x_{i}^{\prime })$ denotes the function *g* applied to the perturbed input $x_{i}^{\prime }$. The input $x_{i}^{\prime }$ is a variation of $x_{i}$ used to evaluate the robustness and performance of the model *g*. The term Ω(g) is a regularization term to control the complexity of *g*.

#### 2.2.2 SHapley Additive exPlanations (SHAP)

SHAP stand as a state-of-the-art explanatory framework, deeply rooted in the foundations of game theory [34] through the utilisation of the Shapley value. This concept provides a systematic and theoretically robust method ensuring a clear understanding of how input features drive model outputs. Through SHAP values, a principled and equitable distribution of influence is secured among the input features, detailing the contribution of each feature towards the differential observed between the actual prediction and collective average prediction across all possible combinations of features. In image analysis, ML or DL model *f* transforms an input *x* (image) into a prediction f(x). The SHAP value $\phi_{i}$ for a feature *i*, and calculate its average impact across every combination of features, represented in Eq. (2) [35].

(2) $$\phi_{i}=\sum_{S\subseteq F\setminus \{i\}}\frac{|S|!(|F|-|S|-1)!}{|F|!}[f_{x}(S\cup \{i\})-f_{x}(S)],$$

where, *F* is the set of all features, and *S* represents the subset of features excluding *i*. The model's prediction is represented as $f_{x}(S)$, when the model considers only the subset *S* of features. Adding feature *i* to this subset changes the prediction to $f_{x}(S\cup \{i\})$, reflects the updated prediction with the feature *i*'s contribution included.

#### 2.2.3 Class Activation Map (CAM)

CAM [36] is a powerful visualisation technique for understanding and diagnosing the behaviour of ML or DL models in medical image analysis, allowing consultants and practitioners to visually assess which area or features within an image are deemed most relevant by the model for a given decision. CAM relies on the CNN architecture with a focus on the activation's within the last convolutional layer. Here, $f_{k}(x,y)$ denotes the activation of unit *k* in the last convolutional layer at spatial position (x,y) and $w_{k}^{c}$ represents the weight corresponding to class *c* for unit *k* in the following fully connected layer, which is replaced by the global pooling layer followed by the output layer in the model using CAM. The CAM for class *c*, denoted as $M_{c}(x,y)$, is formulated as the weighted sum of these last convolutional layer activations.

(3) $$M_{c}(x,y)=\sum_{k}w_{k}^{c}f_{k}(x,y).$$

According to Eq. (3), the contributions of all units *k* in the last convolutional layer to the activation of class *c*, with the weight $w_{k}^{c}$ signifying the relevance of each corresponding feature map $f_{k}$ in classifying the image into class *c*. Consequently, the class activation map $M_{c}$ outlines the critical areas of the image contributing to predicting class *c*, offering a visually interpretable map that highlights the region's most influence on the model's predictions [37].

#### 2.2.4 Gradient Class Activation Mapping (Grad-CAM)

Grad-CAM [38] is one of the most popular XAI methods in image analysis, which improves upon the original CAM by offering a more general approach that can be applied to a wider range of CNNs, including those without a global average pooling layer. Grad-CAM utilises the gradient of any specified target, such as class output, directed towards the last convolutional layer of a CNN to create a localization map highlighting the important region for the target's prediction. To generate the heatmap for class *c*, Grad-CAM calculates the gradient of the class score $y^{c}$ against the feature map $A^{k}$ of the convolutional layer, and then aggregates these gradients over the feature map's dimensions using indices *i* and *j* to drive the significance weights $\alpha_{k}^{c}$ for each neuron. Furthermore, the mentioned weights $\alpha_{k}^{c}$ are computed as follows in Eq. (3):

(4) $$\alpha_{k}^{c}=\frac{1}{Z}\sum_{i}\sum_{j}\frac{\partial y^{c}}{\partial A_{ij}^{k}},$$

here, *Z* represents a normalization factor equivalent to the feature map's total element count, while $\frac{\partial y^{c}}{\partial A_{ij}^{k}}$ denotes the gradient of the class score with respect to each element of the feature map. Finally, the Grad-CAM heatmap $L_{GC}^{c}$ for a target class *c* is generated through a weighted combination of the forward activation maps followed by a ReLU function. This method is designed to ensure that only features with a positive influence on the class of interest are visualised, as shown in Eq. (5) [39].

(5) $$L_{GC}^{c}=\text{ReLU}\left(\sum_{k}\alpha_{k}^{c}A^{k}\right).$$

#### 2.2.5 Guided Grad-CAM (G-Grad-CAM)

G-Grad-CAM [38] is a hybrid XAI approach, providing a fine-grained visual explanation of CNN's decision-making process by combining the concepts of backpropagation and Grad-CAM. In G-Grad-CAM, the visualisation $V_{G-GC}$ for a class *c* can be obtained by element-wise multiplying the maps generated by guided backpropagation and Grad-CAM expressed in Eq. (6).

(6) $$V_{G-GC}=L_{GC}^{c}\circ G,$$

here, $L_{GC}^{c}$ is the heatmap generated by Grad-CAM for class *c*, pinpointing the important region for predicting *c* through weighted gradients. *G* represents the backpropagation map and ∘ denotes the Hadamard product, or element-wise multiplication, used to combine the backpropagation and Grad-CAM heatmaps.

#### 2.2.6 Grad-CAM++

Grad-CAM++ [40] is an updated version of the Grad-CAM method, providing finer visual insights into how CNNs make decisions, particularly effective in images with intricate patterns or numerous occurrences of the same object. This method builds on Grad-CAM by integrating higher-order gradients into its calculations, thereby enabling more precise localization and visualisation of relevant image regions for targeted class predictions. The weights $w_{ij}^{c}$ for class *c* at each pixel (i,j) on the feature map $A^{k}$ are calculated as follows in Eq. (7):

(7) $$w_{ij}^{c}=\frac{\partial y^{c}}{\partial A_{ij}^{k}}\cdot \sigma \left(\frac{\partial^{2}y^{c}}{{\left(\partial A_{ij}^{k}\right)}^{2}}\right)+\sum_{a}\sum_{b}\sigma \left(\frac{\partial^{3}y^{c}}{{\left(\partial A_{ij}^{k}\right)}^{3}}\right)\cdot \frac{\partial y^{c}}{\partial A_{ab}^{k}},$$

where, $y^{c}$ represents the pre-softmax score for class *c*, with the ReLU activation function *σ* used to focus on positive feature influences. It delves into the model's rationale by examining first-order gradients $\frac{\partial y^{c}}{\partial A_{ij}^{k}}$ for immediate influence, second-order gradients $\frac{\partial^{2}y^{c}}{{\left(\partial A_{ij}^{k}\right)}^{2}}$ for capturing non-linear dynamics, and third-order gradients $\frac{\partial^{3}y^{c}}{{\left(\partial A_{ij}^{k}\right)}^{3}}$ to uncover complex feature interactions, providing a layered understanding of how the model predicts class *c*. Subsequently, the localization map $L_{GC++}^{c}$ for class *c* is then calculated by accumulating these weighted activation's throughout all pixels and feature maps, as shown in Eq. (8)

(8) $$L_{GC++}^{c}=\text{ReLU}\left(\sum_{k}\sum_{i}\sum_{j}w_{ij}^{c}\cdot A_{ij}^{k}\right).$$

#### 2.2.7 Saliency map

The saliency map [41] as an XAI method is utilised to illuminate the critical aspects of an input image that impact the CNN's prediction, offering explanations on the model's decision-making process. Mathematically, the creation of the saliency map is based on the gradient of the model prediction score f(x) relative to the input image *x*. Following this, the saliency map *S* is obtained by calculating the gradient $\nabla_{x}f(x)$, which essentially measures the sensitivity of the output score to changes in the input image.

(9) $$S=\left|\frac{\partial f(x)}{\partial x}\right|.$$

According to Eq. (9), the absolute derivative of the model's prediction score per input pixel, highlighting all contributing pixel changes both positive or negative, leads the saliency map to reveal the input image's regions most influential to the model's prediction.

#### 2.2.8 Layer-wise Relevance Propagation (LRP)

LRP [42] is an XAI method that decomposes the output of a DNN back to its input layer, assigning scores to demonstrate each feature's impact on the final decision, thereby offering insight into inputs in contrast to the gradient-based method. In image analysis, LRP allocates the output layer's relevance to the input pixels, navigating backward through the network and calculates the relevance $R_{i}^{\left(l\right)}$ of each neuron *i* in a layer *l*, based on the next layer's l+1 relevance $R_{j}^{\left(l+1\right)}$, connecting weights $w_{ij}$ and activations $x_{i}^{\left(l\right)}$, thus dissecting the pixel-level contributions to the output. The simple LRP rule can be expressed as Eq. (10).

(10) $$R_{i}^{\left(l\right)}=\sum_{j}\frac{w_{ij}x_{i}^{\left(l\right)}}{\sum_{i^{\prime }}w_{i^{\prime }}x_{i^{\prime }}^{\left(l\right)}}R_{j}^{\left(l+1\right)}.$$

#### 2.2.9 Surrogate model

The surrogate model [43] in XAI refers to the approach that approximates the functionality of complex ML or DL models utilised in image processing. This method explains how input image pixels affect predictions, making it invaluable for comprehending and explaining the complex model's decision-making process. Mathematically, on the given input image *x*, the complex model produces output f(x) and g(x) is the corresponding output from the surrogate model. The loss function *L* is utilised to minimise the difference between f(x) and g(x) for all input images. The overall procedures are presented in Eq. (11).

(11) $$L(f,g)=\sum_{x\in X}{\left\|f(x)-g(x)\right\|}^{2},$$

where, the set of input images *X* with the chosen loss function *L*, typically mean squared error, evaluates the difference between the outputs from the complex model f(x) and the surrogate model g(x). The surrogate model *g* is trained to minimise the loss, making its prediction as close as possible to that of the complex model *f*.

#### 2.2.10 Integrated Gradient (IG)

IG [44] is an XAI approach that offers a way to attribute the prediction of ML or DL models to its input features, notably pixels for images by integrating the output gradients from a baseline to the actual image; thereby highlighting the role of an individual pixel in image analysis. Consider a given input image *x* and a baseline image $x^{\prime }$ with the Integrated Gradients IG along the *i*-th dimension for an input feature $x_{i}$ as defined in Eq. (12).

(12) $$IG_{i}(x)=(x_{i}-x_{i}^{\prime })\times \int_{\alpha =0}^{1}\left(\frac{\partial f(x^{\prime }+\alpha \times (x-x^{\prime }))}{\partial x_{i}}\right)d\alpha ,$$

where f(x) represents the model's output for the input *x*, and $\frac{\partial f(x)}{\partial x_{i}}$ is the gradient of f(x) with respect to the input feature $x_{i}$. The parameter *α* is utilized to scale the interpolation path from the baseline image $x^{\prime }$ to the input image *x*. Meanwhile, $x_{i}-x_{i}^{\prime }$ amplifies the IG based on the variance of each feature from the baseline, focusing on their contributions to the model's decision.

#### 2.2.11 Counterfactual explanation

The counterfactual explanation [45] is one of the popular methods in XAI that provides insights into model decisions by addressing “what-if” questions and identifying the minimal transformation required to change a model's output. From a mathematical perspective, given an original input image *x* and the model *f* that outputs the decision f(x), the counterfactual explanation aims to discover an alternate image $x^{\prime }$ that is as close as possible to *x* but leads to a different and predefined decision $f(x^{\prime })\neq f(x)$. Subsequently, to minimise the difference between *x* and $x^{\prime }$ while ensuring that $x^{\prime }$ changes the model's decision [46].

(13) $$\min D(x,x^{\prime })+\lambda L(f(x^{\prime }),y^{\prime })\;\text{subject to}\;x^{\prime }\in X,f(x)\neq f(x^{\prime }),$$

where, the function $D(x,x^{\prime })$ quantifies the distance between the original *x* and counterfactual $x^{\prime }$ images, aiming for minimal deviation to maintain similarity. $L(f(x^{\prime }),y^{\prime })$ represents the loss function, gauges how well the counterfactual prediction $f(x^{\prime })$ matches a chosen outcome $y^{\prime }$ differing from the original model's output f(x). The regularization parameter *λ* balances the importance of minimising the distance $D(x,x^{\prime })$ against achieving the desired outcome $L(f(x^{\prime }),y^{\prime })$, while *X* signifies the domain of all possible images. Lastly, $f(x)\neq f(x^{\prime })$ as a prerequisite ensures the counterfactual diverges from the original decision, central to crafting effective counterfactual.

#### 2.2.12 Occlusion Analysis (OA)

In XAI approaches, the OA [47] is a method that evaluates how occluding areas of an image affect the model's decision. This method masks regions of an image with a uniform patch to observe how the model's output changes. In occlusion analysis, a model *f* generates a prediction score f(x) for an image *x*. Following this, an occluded version of the image $x_{\text{occ}}$ is created by masking a region of the image, and then the prediction score for this image is evaluated as $f(x_{\text{occ}})$. The significance of the masked area is determined by comparing the prediction scores for *x* and $x_{\text{occ}}$.

(14) $$I_{\text{region}}=f(x)-f(x_{\text{occ}}),$$

here, in Eq. (14), $I_{\text{region}}$ expresses the importance of the masked region, where a larger difference indicates a higher importance of the occluded region in influencing the model's decision.

#### 2.2.13 Randomized Input Sampling for Explanation (RISE)

In XAI, RISE [48] utilises random masking to determine the impact of specific image areas on model predictions, applying diverse masks that obscure sections of the input. In the RISE framework, given an input image (Un-masked) *x* and its model prediction f(x), the utilised series of randomly generated binary masks are *M*. Each mask m∈M is applied to the *x* to create a masked version of the image $x_{m}=x⊙m$, here ⊙ represents the element-wise multiplication. The model's predictions are then computed for these masked images, leading to a series of prediction scores $f(x_{m})$. Subsequently, for every pixel *i* the importance score $S_{i}$ is determined by calculating the average effect of all masks on the model's prediction, weighted by the pixel's visibility within those masks, expressed in Eq. (15).

(15) $$S_{i}=\frac{1}{\left|M\right|}\sum_{m\in M}m_{i}\cdot f(x_{m}),$$

where, $m_{i}$ indicates the status of the mask *m* at pixel *i*. Here, $m_{i}=1$ means the pixel *i* is visible, and $m_{i}=0$ means the pixel *i* is hidden. |M| represents the total number of masks used.

#### 2.2.14 Permutation Importance (PI)

PI [49], also known as feature importance as an XAI approach, evaluates the impact of features such as pixels in images on model performance by shuffling these features across the dataset and noting performance changes. In image processing, this method shuffles pixel values or regions among images to identify their contribution to model prediction, with a notable decrease in performance highlighting the feature's significance. In PI, the process starts by considering the model's prediction f(x) and loss function L(y,f(x)) that evaluates the difference between predictive value and actual value *y* for an input image *x*. The baseline performance $P_{\text{bl}}$ is calculated as the average loss across all *N* images in the dataset, where $y_{n}$ is the actual label and $x_{n}$ is the *n*-th image.

(16) $$P_{\text{bl}}=\frac{1}{N}\sum_{n=1}^{N}L(y_{n},f(x_{n})).$$

The performance for a new dataset $P_{\text{shuff},i}$ is then determined by calculating the average loss for images with the *i*-th pixel pixel-shuffled, denoted as $x_{n,i}^{\prime }$ for the *n*-th image.

(17) $$P_{\text{shuff},i}=\frac{1}{N}\sum_{n=1}^{N}L(y_{n},f(x_{n,i}^{\prime })).$$

The PI $I_{i}$ of pixel *i* is derived by:

(18) $$I_{i}=P_{\text{shuff},i}-P_{\text{bl}},$$

where positive $I_{i}$ values indicate a reduction in model performance due to the shuffling of pixel *i*, highlighting its significance in the model's decision-making performance.

#### 2.2.15 Morris Sensitivity Analysis (MSA)

In XAI, MSA [50] provides insights into how variation in input features affect model decisions, highlighting the most and least influential inputs and their interactions. In image analysis, the MSA rigorously alters the values of individual pixels or clusters of pixel groups to evaluate their impact on predictive outcomes. This method entails generating a baseline input, followed by the formulation of a sequence of modified input sets. Each set varies a single input feature from the baseline, allowing for a thorough investigation into the contribution of specific features to predictive performance. Image analysis in MSA starts with a baseline input vector *x* by representing the original pixel values of an image. Subsequently, for each feature *i* with perturbed input vector $x_{i}^{\prime }$ generates from the *x* by altering the *i*-th feature's values through a predefined amount Δ, while keeping other features constant. Next, the model prediction is evaluated for the original input f(x), and altered input $f(x_{i}^{\prime })$. The impact of altering feature *i* on the model's output is measured through elementary effect $EE_{i}$, presented in Eq. (19).

(19) $$EE_{i}=\frac{f(x_{i}^{\prime })-f(x)}{\Delta }.$$

#### 2.2.16 Gradient Attention Rollout (GAR)

GAR [51] is an XAI method that combines gradient data and attention weights, providing insights into how DNNs process input features (image pixels) through the model's layers. This approach effectively highlights the pathways that contribute most significantly to the model's decision. In the context of image analysis, the GAR applies attention maps and output gradients to illustrate the model's process of weighing and merging various parts across layers for its final decision. GAR starts by identifying attention weights $A^{\left(l\right)}$ in each layer *l*, where $A_{ij}^{l}$ indicates the attention from the feature *i* to *j*, calculating the output's gradients relative to these weights $\nabla A^{\left(l\right)}$ to understand their impact on prediction. Finally, for each layer *l*, the rollout value $R^{\left(l\right)}$ is computed as shown in Eq. (20).

(20) $$R^{\left(l\right)}=\prod_{k=l}^{L}(A^{k}⊙\nabla A^{k}),$$

where, *L* represents the final layer and ⊙ denotes element-wise multiplications. This process integrates attention and gradient data, offering insights into how initial features contribute to the model's decision.

#### 2.2.17 Attention-based method

Attention-based [52] XAI approach in image analysis leverages the attention mechanisms within CNNs or transformers to explain how models make decisions by pinpointing the crucial area of an image for prediction. These mechanisms assign importance weights to each part of the image, indicating their significance in the model's final decision. In this method, the process begins with the computation of attention weights for features $X=\{x_{1},x_{2},\ldots ,x_{n}\}$, where $x_{i}$ representing the corresponding vector to different segments of the image. These weights $W=\{w_{1},w_{2},\ldots ,w_{n}\}$ are derived through an attention-function $f_{\text{attention}}$ followed by a SoftMax to ensure the weights are normalized as expressed in Eq. (21).

(21) $$W=\text{softmax}(f_{\text{attention}}(X)).$$

Subsequently, the attention-based image representation *A* is derived from this $A=\sum_{i=1}^{n}w_{i}\cdot x_{i}$ and then the model uses *A* for prediction.

#### 2.2.18 Ablation Studies (AS)

In image analysis, AS [53] within XAI systematically manipulate or eliminate particular model components including pixels, convolutional layers, or neurons, to evaluate their effect on model output. This approach illuminates the role and impact of different model components on the decision-making process. In the AS framework, the model's prediction f(x) for an input image *x* contains specific image features; altering these features to form $x^{\prime }$ changes the output to $f^{\prime }(x^{\prime })$. Following this, the effect of ablation is quantified by comparing the model's performance metrics before and after the ablation process. The impact *I* is defined in Eq. (22).

(22) $$I=\text{Acc}(f(x))-\text{Acc}(f^{\prime }(x^{\prime })).$$

#### 2.2.19 Deep Taylor Decomposition (DTD)

DTD [54] in XAI provides a framework that maps out how input features (image pixels) contribute to a model's prediction, applying DTD principles for DNNs application. This approach is especially useful for understanding which parts of an input image are most influential in the model's decision-making process. For a given input image *x*, the model's output f(x) with the goal is to decompose f(x) into relevance score $R_{i}$ for each input pixel *i*. Next, starting from the output, the relevance scores are traced back to the input layer with each neuron *j* in layer l+1 passes $R_{j}^{\left(l+1\right)}$ to preceding neurons *i* in layer *l*, factoring in their contribution. This process predominantly utilizes the connection weights $w_{ij}$ and the activation $a_{i}^{\left(l\right)}$ of neurons *i* facilitates the redistribution. Following this, the relevance score $R_{i}^{\left(l\right)}$ for neuron *i* in layer *l* is calculated in Eq. (23).

(23) $$R_{i}^{\left(l\right)}=\sum_{j}\left(w_{ij}\cdot a_{i}^{\left(l\right)}\right)R_{j}^{\left(l+1\right)}.$$

### 2.3 Medical images

Medical imaging plays essential role in contemporary diagnostic and treatment planning, offering detailed visual insights into the human body's internal structures. Each imaging method provides distinct benefits for the detection, diagnosis and monitoring of wide range of medical conditions. Following, we briefly introduce five key imaging modalities which are utilised in the considered publications.

• **Fundus Images:** Fundus imaging captures detailed photographs of the eye's interior, essential for diagnosing conditions like diabetic retinopathy, glaucoma, and macular degeneration.
• **Endoscopy:** Endoscopy uses a flexible tube with a camera to view internal organs and cavities, aiding in diagnosing gastrointestinal, respiratory, and other hollow organ conditions.
• **X-rays:** X-rays produce images of the body's interior, especially bones, and are crucial for identifying fractures, infections, and certain diseases like pneumonia and cancers.
• **Magnetic Resonance Imaging (MRI):** MRI uses magnetic fields and radio waves to create detailed images of soft tissues, aiding in the diagnosis of brain tumours, spinal injuries, and musculoskeletal disorders.
• **Computed Tomography (CT) Scans:** CT scans use X-ray measurements and computer processing to generate detailed cross-sectional images, vital for diagnosing cancers, cardiovascular diseases, and trauma.

## 3 Methodology

In this section, we provide an overview of the methodology employed in designing this systematic literature review.

### 3.1 Literature review design

Our systematic literature review methodology comprises three distinct phases: (i) active planning, (ii) conducting and reporting the review results, and (iii) exploration of research challenges according to the widely accepted guidelines and processes outlined in [55] and [56], [57]. The research questions, the identification process for this study, and the data extraction procedures are covered in detail in the remaining section.

### 3.2 Research questions

The goal of this survey is to offer a comprehensive overview of the extant literature that provides a discussion on XAI, encompassing various methodologies, performance metrics, its role in disease diagnosis, and its broader applications within medical imaging. The defined research questions are as follows:

1. What XAI approaches have been utilised for medical image analysis?
2. In medical imaging, for which particular diseases do XAI techniques enhance the explainability and confidence of AI-based diagnoses?
3. What are the evaluation metrics that are used to assess the effectiveness of XAI applications in medical imaging?
4. What are the strengths, weaknesses, limitations, and future research directions of XAI methods?

### 3.3 Identification of research

**Fig. 2:**
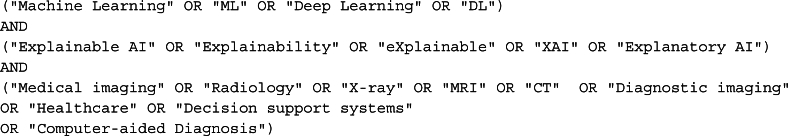
Search string.

Literature was sourced from four prominent electronic databases: (i) IEEE Xplore, (ii) Web of Science, (iii) PubMed, and (iv) ACM Digital Library. The search string utilised for querying these databases based on metadata attributes, including title, abstract, and keywords, is summarised in Fig. 2.

**Fig. 3:**
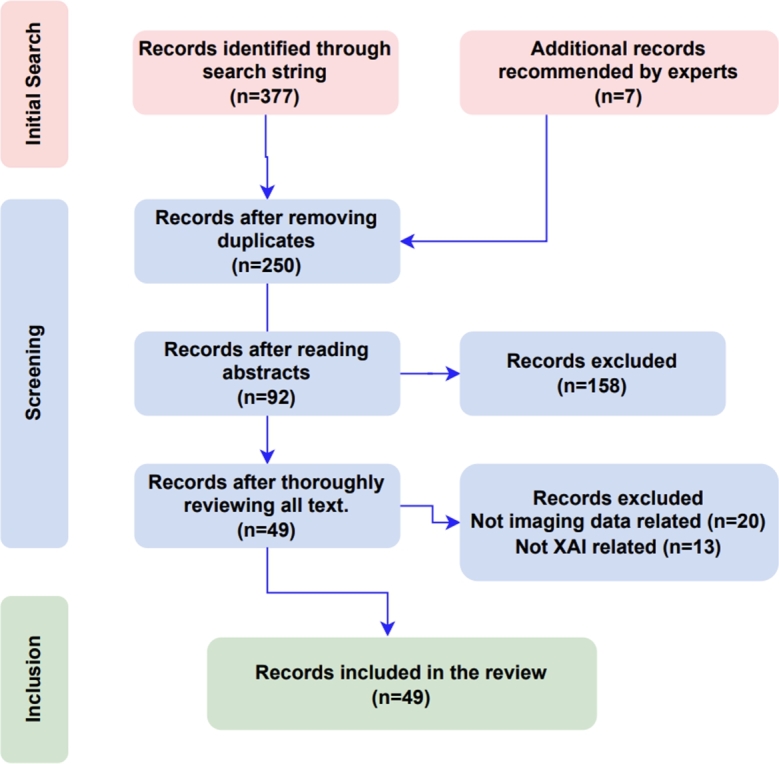
Flow diagram of our review, it shows the number of studies identified, screened and included in this review.

A total of 377 publications relevant to the research topic were identified in the initial search. A set of inclusion and exclusion criteria Table 1 was established to ensure a systematic and replicable selection, as presented in the flow diagram (Fig. 3) of our review, which shows the number of studies identified, screened and included in this review. In addition, a selection of carefully chosen publications [58], [59], [60], [61], [62], [63], [64] that were recommended by research topic experts but were not found by the search string were also included.

**Table 1:** Criteria for inclusion and exclusion.

Inclusion Criteria	Exclusion Criteria
• Full text available • Published during 2015 to 2023 • Published in the considered databases • Work published in workshops (W), symposiums (S), conferences (C), books (B), and journals (J) across all disciplines • English-language papers exploring the definition, explanation, methodologies, approaches, evaluation metrics, image analysis, image processing, disease diagnosing, and the role of Explainable Artificial Intelligence (XAI) in healthcare	• Uncompleted studies • Not in English • Duplicated papers • Studies that discuss XAI in image analysis beyond the realm of medical imaging

The researchers independently removed duplicates and then screened the titles in accordance with the recommendations of [56], [57], which led to the deletion of papers and a reduction in the count to 250. Subsequent reading of abstracts further narrowed the selection to 92 papers. In the final phase, all texts were thoroughly reviewed, and any disagreements over which paper should be included were discussed and resolved until an agreement was reached. The accompanying Table 2 lists 49 research publications that were considered appropriate for inclusion in the final review, based on these criteria provided in Table 1.

**Table 2:** Selection of final publications. Key: Journal Article - J, Conference Paper - C.

No	Paper Title	Authors	Type	Year	Venue	Citations	Rank
1	Deep-learning-assisted diagnosis for knee magnetic resonance imaging: Development and retrospective validation of MRNet.	Bien et al.	J	2018	Plos Medicine	580	Q1
2	Layer-wise relevance propagation for explaining deep neural network decisions in MRI-based Alzheimer's disease classification	Bohle et al.	J	2019	Aging Neuroscience	236	Q2
3	Explainable AI and Mass Surveillance System-Based Healthcare Framework to Combat COVID-19 Like Pandemics	Hossain et al.	J	2020	IEEE Network	322	Q1
4	EXAM: An Explainable Attention-based Model for COVID-19 Automatic Diagnosis	Shi et al.	C	2020	11th ACM Int. Conf. on Bioinformatics Computational Biology and Health Informatics	19	C
5	A Proposal for an Explainable Fuzzy-based Deep Learning System for Skin Cancer Prediction	Lima et al.	C	2020	Int. Conf. on eDemocracy, eGovernment (ICEDEG)	12	N/A
6	Assessment of knee pain from MR imaging using a convolutional Siamese network	Chang et al.	J	2020	European Radiology	50	Q1
7	Volumetric breast density estimation on MRI using explainable deep learning regression	Velden et al.	J	2020	Scientific Report (Nature)	40	Q1
8	Demystifying brain tumour segmentation networks: interpretability and uncertainty analysis	Natekar et al.	J	2020	Computational Neuroscience	77	Q3
9	Clinical Interpretable Deep Learning Model for Glaucoma Diagnosis	Liao et al.	J	2020	IEEE Journal of Biomedical and Health Informatics	98	Q1
10	An interpretable classifier for high-resolution breast cancer screening images utilizing weakly supervised localization	Shen et al.	J	2020	Medical Image Analysis	152	Q1
11	Explainable Data Analytics for Disease and Healthcare Informatics	Leung et al.	C	2021	25th Int. Conf. on Database Systems for Advanced Applications	22	C
12	Doctor's Dilemma: Evaluating an Explainable Subtractive Spatial Lightweight Convolutional Neural Network for Brain Tumor Diagnosis	Kumar et al.	J	2021	ACM Transactions on Multimedia Computing, Communications, and Applications	15	Q1
13	Prediction of Quality of Life in People with ALS: On the Road Towards Explainable Clinical Decision Support	Antoniadi et al.	J	2021	ACM SIGAPP Applied Computing Review	5	N/A
14	Predicting the Evolution of Pain Relief: Ensemble Learning by Diversifying Model Explanations	Costa et al.	J	2021	ACM Transactions on Computing for Healthcare	2	Q2
15	xViTCOS: Explainable Vision Transformer Based COVID-19 Screening Using Radiography	Mondal et al.	J	2021	IEEE Journal of Translational Engineering in Health and Medicine	16	Q2
16	An Explainable System for Diagnosis and Prognosis of COVID-19	Lu et al.	J	2021	IEEE Internet of Things Journal	10	Q1
17	Using Causal Analysis for Conceptual Deep Learning Explanation	Single et al.	C	2021	Medical Image Computing and Computer Assisted Intervention – MICCAI 2021	18	A
18	Explainable Predictions of Renal Cell Carcinoma with Interpretable Tree Ensembles from Contrast-enhanced CT Images	Han et al.	C	2021	International Joint Conference on Neural Networks (IJCNN)	0	B
19	An algorithmic approach to reducing unexplained pain disparities in underserved populations	Pierson et al.	J	2021	Nature Medicine	202	Q1
20	Explainable multi-instance and multi-task learning for COVID-19 diagnosis and lesion segmentation in CT images	Li et al.	J	2022	Knowledge-Based Systems	24	Q1
21	Comparative analysis of explainable machine learning prediction models for hospital mortality	Stenwig et al.	J	2022	BMC Medical Research Methodology	27	Q1
22	Towards an Explainable AI-based Tool to Predict the Presence of Obstructive Coronary Artery Disease	Kokkinidis et al.	C	2022	26th Pan-Hellenic Conference on Informatics	1	N/A
23	Tuberculosis detection in chest radiograph using convolutional neural network architecture and explainable artificial intelligence	Nafisah and Muhammad	J	2022	Neural Computing and Applications	48	Q1
24	Fairness-related performance and explainability effects deep learning models for brain image analysis	Stanley et al.	J	2022	Journal of Medical Imaging	14	Q2
25	Automating Detection of Papilledema in Pediatric Fundus Images with Explainable Machine Learning	Avramidis et al.	C	2022	IEEE International Conference on Image Processing (ICIP)	5	B
26	Towards Trustworthy AI in Dentistry	Ma et al.	J	2022	Journal of Dental Research	23	Q3
27	GANterfactual—Counterfactual Explanations for Medical Non-experts Using Generative Adversarial Learning	Mertes et al.	J	2022	Frontiers in Artificial Intelligence	42	Q2
28	An Explainable Medical Imaging Framework for Modality Classifications Trained Using Small Datasets	Trenta et al.	C	2022	International Conference on Image Analysis and Processing	3	N/A
29	The effect of machine learning explanations on user trust for automated diagnosis of COVID-19	Goel et al.	J	2022	Computers in Biology and Medicine	25	Q1
30	Detection of COVID-19 in X-ray Images Using Densely Connected Squeeze Convolutional Neural Network (DCSCNN): Focusing on Interpretability and Explainability of the Black Box Model	Ali et al.	J	2022	Sensors	6	Q1
31	Explainable AI for Glaucoma Prediction Analysis to Understand Risk Factors in Treatment Planning	Kamal et al.	J	2022	IEEE Transactions on Instrumentation and Measurement	32	Q1
32	Explanation-Driven HCI Model to Examine the Mini-Mental State for Alzheimer's Disease	Loveleen et al.	J	2023	ACM Transactions on Multimedia Computing Communications and Applications	23	Q1
33	Interpretable Models for ML-based Classification of Obesity	Khater et al.	C	2023	7th International Conference on Cloud and Big Data Computing	1	N/A
34	Directive Explanations for Monitoring the Risk of Diabetes Onset: Introducing Directive Data-Centric Explanations and Combinations to Support What-If Explorations	Bhattacharya et al.	C	2023	28th International Conference on Intelligent User Interfaces	7	A
35	VR-LENS: Super Learning-based Cybersickness Detection and Explainable AI-Guided Deployment in Virtual Reality	Kundu et al.	C	2023	28th International Conference on Intelligent User Interfaces	0	A
36	Ante- and Post-Hoc Explanations for Prediction Models of Cisplatin-Induced Acute Kidney Injury: A Comparative Study	Nishizawa et al.	C	2023	7th International Conference on Medical and Health Informatics	0	N/A
37	Improving explainable AI with patch perturbation-based evaluation pipeline: a COVID-19 X-ray image analysis case study	Sun et al.	J	2023	Scientific Reports (Nature)	2	Q1
38	Explainable deep learning-based clinical decision support engine for MRI-based automated diagnosis of temporomandibular joint anterior disk displacement	Yoon et al.	J	2023	Computer Methods and Programs in Biomedicine	5	N/A
39	Deep learning referral suggestion and tumour discrimination using explainable artificial intelligence applied to multiparametric MRI	Shin et al.	J	2023	European Radiology	4	Q1
40	Automated prediction of COVID-19 severity upon admission by chest X-ray images and clinical metadata aiming at accuracy and explainability	Olar et al.	J	2023	Scientific Reports (Nature)	0	Q1
41	Explaining the black-box smoothly—A counterfactual approach	Singla et al.	J	2023	Medical Image Analysis	45	Q1
42	An Intelligent Thyroid Diagnosis System Utilising Multiple Ensemble and Explainable Algorithms with Medical Supported Attributes	Sutradhar et al.	J	2023	IEEE Transactions on Artificial Intelligence	2	Q1
43	Explainable Artificial Intelligence (XAI) for Deep Learning Based Medical Imaging Classification	Ghnemat et al.	J	2023	Journal of Imaging	4	Q2
44	An Explainable AI System for Medical Image Segmentation With Preserved Local Resolution: Mammogram Tumor Segmentation	Farrag et al.	J	2023	IEEE Access	1	Q1
45	Wireless Capsule Endoscopy Image Classification: An Explainable AI Approach	Varam et al.	J	2023	IEEE Access	1	Q1
46	An Explainable Brain Tumor Detection Framework for MRI Analysis	Yan et al.	J	2023	Applied Sciences	4	Q2
47	Explainable AI for Retinoblastoma Diagnosis: Interpreting Deep Learning Models with LIME and SHAP	Aldughayfiq et al.	J	2023	Diagnostics	12	Q2
48	NeuroXAI++: An Efficient X-AI Intensive Brain Cancer Detection and Localization	Rahman et al.	C	2023	International Conference on Next-Generation Computing, IoT, and Machine Learning (NCIM 2023)	3	N/A
49	Lung Cancer Detection Using Deep Learning and Explainable Methods	Alomar et al.	C	2023	International Conference on Information and Communication Systems (ICICS)	0	C

### 3.4 Data extraction

The papers were manually reviewed independently by researchers. Bibliographic information and contributions to the domain of XAI in medical imaging, including machine learning/deep learning models, XAI methods and approaches, datasets used, image modality, disease diagnosis, and evaluation metrics used, were extracted for each of the 49 papers. Subsequently, the retrieved data were compared and meticulously aligned with in-depth conversations to resolve disagreements and contradictions.

## 4 Results

The returned papers were categorized based on the XAI methods applied to medical image analysis. This section discusses both the preliminary and detailed analysis of this categorization.

### 4.1 Preliminary analysis

**Fig. 4:**
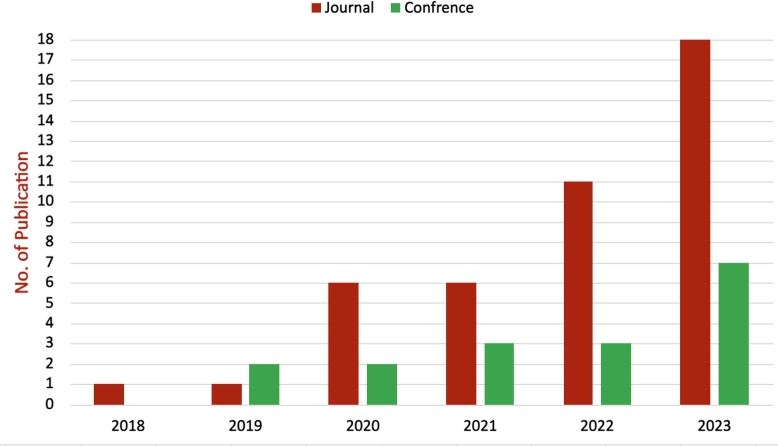
No. of publications per year.

**Fig. 5:**
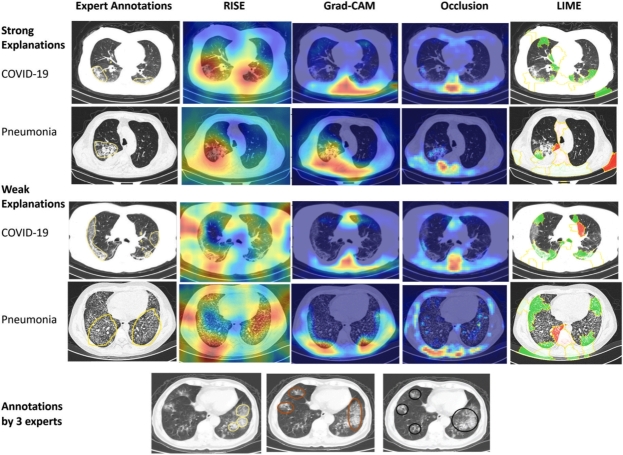
RISE, Grad-CAM, OA and LIME explanations by [65], display human annotations and explanations generated by mentioned methods for a COVID-19 CT image. Each explanation technique highlights salient regions responsible for the prediction. Human annotations highlight different salient regions. In the generated explanations, red regions indicate areas contributing to the prediction when using RISE, Grad-CAM, and OA. LIME differentiates pixels supporting the prediction in green and those negating the prediction in red.

Fig. 4 (extracted from Table 2) offers an insightful glimpse of the changing landscape of published research work in the realm of XAI in medical imaging between 2015 to 2023,^2^ breaking down the output into conference, journal and survey papers. The statistics suggest a thriving interest and acceptance of the XAI in health within academic circles, journal papers started with a minimal presence in 2018 and exhibit a generally increasing trend in publications over the years. Compared to journal submissions, conference papers show more variability year over year with a noticeable jump in 2021 followed by an even greater peak in 2022. This marked growth could indicate a period of integration within the field, where the research community is synthesising the information and establishing a comprehensive understanding of the current spectrum of XAI in medical imaging. According to Table 2, the majority of the considered publications are in Q1 journals, with many others in Q2 journals. Similarly, the conference papers are presented at prestigious conferences ranked A, B, and C. These venues suggest that XAI for medical imaging is a popular and well-accepted topic among researchers in reputable journals. Additionally, 44 out of 49 papers are cited by researchers in their technical works, indicating a strong interest in utilising XAI for medical images analysis and healthcare applications (Fig. 5).

### 4.2 LIME for medical images

This section summarises the papers that used LIME as the XAI method.

The authors of [66] used the densely connected squeeze CNNs for COVID-19 classification using four datasets. The authors implemented LIME to visualise the attention region in the image and decision of the model, thereby improving trust, transparency and explainability. Following this, the VGG-16 was used for the COVID-19 classification and reviewed their model predictions through LIME, aiming to enhance trust in complex architecture [67]. Similarly, to evaluate the CNNs prediction's decision for common Pneumonia, CT and X-ray images were explained through LIME by [65], [64]. Another framework, Generative Adversarial Networks (GANs) with the implementation of LIME was presented by [61] for Pneumonia detection in the X-ray images. Furthermore, an ML-based Thyroid disease prediction system was proposed by [68], which can potentially predict the disease by considering three feature selection techniques such as feature importance, information gain selection and lease Absolute Shrinkage and selection operator to reduce the dimension of the dataset. The authors applied LIME to explain the reasons behind the decision of the proposed system. The researchers of [62] presented an adaptive neuro-fuzzy inference system (ANFIS) and pixel density analysis (PDA) for Glaucoma predictions from infected and healthy fundus images and employed LIME to provide trustworthy explanations. Subsequently, various DL architectures including vision transformer were trained on the Kvasir-capsule image dataset for Gastrointestinal identification from endoscopy images. Varam et al. [69] applied LIME to compare and analyse their performance through LIME explanations. Furthermore, the authors of [70] introduced an explainable HCI model using the LIME and ML techniques to identify Alzheimer's disease in MRI images and explain the model decision-making process. Aldughayfiq et al. [58] utilised DNN for Retinoblastoma diagnosis from fundus images and explored the use of LIME to generate the local explanations for the proposed model. Further, in [71], Inceptionv3 and ResNet50 were implemented to accurately detect chronic lung cancer in CT images. The researchers utilised LIME to provide insights into the decision-making process of the employed architectures.

LIME provides easily understandable explanations by highlighting influential features (image pixels) in individual predictions, making complex diagnostic models more explainable to clinicians. However, the reliability of LIME's explanations may suffer from the inconsistencies across various runs and changes in input, due to its reliance on local approximations and perturbation strategies. Moreover, while providing valuable local insights, LIME can be computationally intensive and might not reflect the overall behaviour of the model across broader imaging. Additionally, the researcher did not employ any evaluation metrics to measure the performance of their explainability using LIME.

### 4.3 SHAP for medical images

**Fig. 6:**
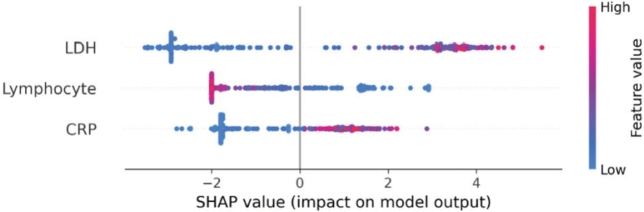
SHAP explanations by [72], illustrated feature importance using SHAP values. Each row in the figure represents a different feature, while each point corresponds to a sample. The colour gradient indicates the value of the feature: redder points signify larger values, while bluer points represent smaller values. In the context of mortality prediction, treated as a binary classification problem where 1 indicates death, the figure shows several red points on the right side of the SHAP values for features like CRP and LDH, suggesting that higher values of these features are associated with an increased risk of mortality. Conversely, for the lymphocyte feature, blue points are concentrated on the right, indicating that lower lymphocyte levels are linked to higher mortality. Overall, the figure demonstrates that elevated levels of LDH and CRP, along with reduced lymphocyte levels, are associated with a higher likelihood of death.

This section summarises the work that used SHAP as the XAI method for medical image analysis (Fig. 6).

Leung et al. [73] presented an explainable data analytics system for COVID-19 and healthcare informatics consisting of a predictor and explainer component. In the predictor component, the RF and NN-based few-shot models were implemented to make predictions from the historical data, while in the explainer component, SHAP was used to provide explanations for specific instances by showing how feature values contribute to positive or negative predictions. Similarly, in Wuhan China, a data-driven medical assistance system was designed by [72] using ML and DL approaches to diagnose and predict the prognosis of COVID-19. Further, the authors of [70] introduced an explainable HCI model using the SHAP and ML approaches to identify Alzheimer's disease in MRI images and explain the model decision-making process. Subsequently, a Clinical decision support system (CDSS) was designed for Amyotrophic lateral sclerosis (Motor neuron) disease, to alert the clinician when patients are at risk of experiencing low quality of life. The authors employed XGBoost with the SMOTE technique for prediction and explained the contributory features to the model via SHAP [74]. To predict the pre-test probability of stable Coronary artery disease (CAD) using various ML algorithms is developed by [75]. This study focused on providing interpretable explanations to clinicians using SHAP to increase acceptance of the models. The researchers of [76] designed an explanation dashboard that predicts the risk of diabetes onset and employed the SHAP to explain the important features of the model's decision. Moreover, the ensemble ML models were trained on three different datasets to detect cybersickness and chronic pain by [77], [78]. The authors utilised SHAP to explain the model output and identify the dominant features. A comparative study was conducted by [79], and multiple ML algorithms were applied for accurate prediction of Cisplatin-induced kidney injury (Cis-AKI) using patient data between 2006 to 2013. The performance of these methods was evaluated through SHAP to explain which model is accurate and understandable. Additionally, to predict Renal cell carcinoma using CT images by [80], proposed a Tree ensemble-based model with four strategies: multiscale feature extraction, attribute optimization, SHAP for interpretation and the decision curve analysis for clinical utility evaluation. Van et al. [81] demonstrated the feasibility of automatically estimating volumetric breast density in MRI images without the need for segmentation, utilised 3-dimensional regression CNN and integrated with SHAP. Following this, diverse deep learning architectures, such as the vision transformer, were utilised to train on the Kvasir-capsule image dataset to identify gastrointestinal features from endoscopy images. [69] employed SHAP for performance comparison and analysis through explanations generated by SHAP. Further, the authors of [58] utilised DNN to diagnose Retinoblastoma from fundus images, incorporating SHAP to produce interpretive explanations for the model's output.

SHAP uses Shapley values from game theory to provide a solid and rigorous method for attributing the impact of individual features such as pixel intensity, colour, and textures on medical images on model output, ensuring that explanations are both fair and consistent across different prediction instances. On the other hand, the computational requirements for SHAP are substantial for high-dimensional medical images, where the feature space can include thousands of pixels or voxel elements, making it hard to use it in real-time diagnostic settings. Additionally, the SHAP explanations provided are detailed but can be complex to understand and interpret, which might make it difficult for medical professionals. Furthermore, the researcher did not utilize any evaluation metrics to assess the performance of SHAP.

### 4.4 CAM for medical images

**Fig. 7:**
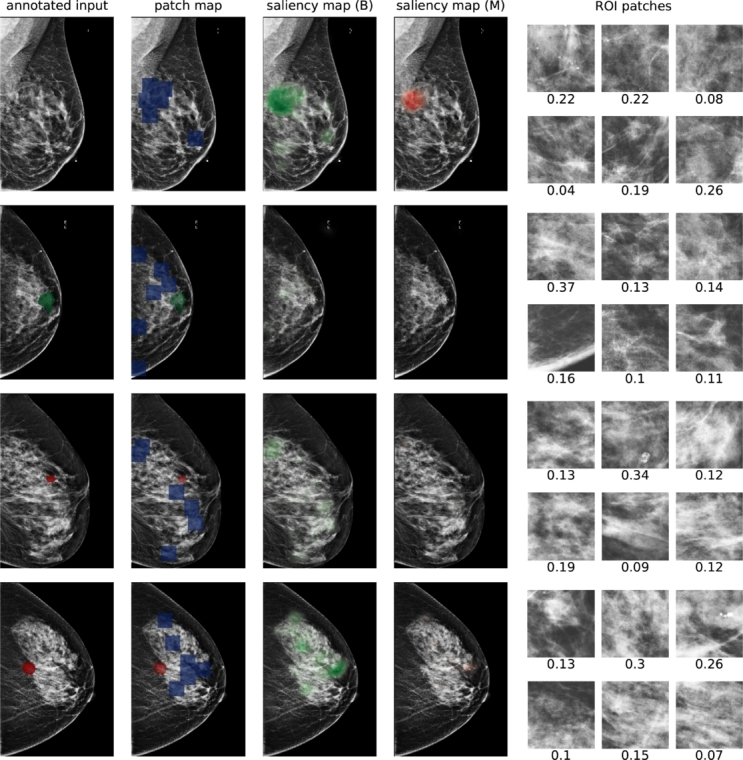
CAM visualization utilizing the Saliency map by [82], illustrates results for four examples, showing annotated input images, ROI patches, saliency maps for benign and malignant classes, and ROI patches with attention scores. The top example features a circumscribed oval mass in the left upper breast middle depth, diagnosed as a benign fibroadenoma via ultrasound biopsy. The second example displays an irregular mass in the right lateral breast posterior depth, diagnosed as invasive ductal carcinoma via ultrasound biopsy. The third example's saliency maps identify benign findings: a circumscribed oval mass confirmed as a benign fibroadenoma, a smaller oval mass recommended for follow-up, and an asymmetry that is stable and benign. The bottom example shows segmental coarse heterogeneous calcifications in the right central breast middle depth, diagnosed as high-grade ductal carcinoma in situ via stereotactic biopsy.

An explainable DL-based model was proposed by [83], aimed at delivering a reliable tool for medical professionals in the diagnosis of Brain tumours, while also enhancing the model's performance. The authors developed and trained the Subtractive Spatial Lightweight CNN (SSLW-CNN) using MRI images and the model's evaluation was conducted using CAM visualization to provide insights from an XAI standpoint. Moreover, Sandford University's medical data comprising MRI images, was employed to identify Knee injuries using DNN. CAM was utilised to present model predictions to clinicians, aiding the diagnostic imaging process [84]. Similar to the preceding work, the authors of [85] presented the application of a Convolutional Siamese network to link MRI scans of an individual's knees experiencing unilateral knee pain. CAM was applied to elucidate the model's decision-making process. Following this, Bohle et al. [86] used the ML approach to devise a novel algorithmic method for assessing the severity of Osteoarthritis using knee radiographs. They proposed an Algorithmic Severity Score (ALG-P) aimed at distinguishing between two hypotheses. The study found that the ALG-P score better predicts pain severity than the Kellgren-Lawrence grade. To demonstrate the predictive accuracy of their model in a manner that supports explainable and responsible AI, they utilised the CAM approach. Furthermore, an interpretable NN model was proposed by [82], specifically tailored to the distinctive characteristics of Breast cancer X-ray images. This model applies a low-capacity network to pinpoint informative regions followed by a high-capacity network to extract actual features from those identified regions. The authors assessed the model predictions using the CAM method. Yan et al. [59] introduced an explainable framework for brain tumour detection that encompasses segmentation, classification and explanation tasks. This framework integrates two streamlined and effective CNNs to thoroughly examine MRI images of Brain tumours and explain the model outcomes using CAM. In addition, the authors of [70] proposed a Double-detailed CNN module that preserves local spatial resolution while enlarging the receptive field for tumour image segmentation tasks. The mentioned approach overcomes the limitation of detailed convolutional, which may result in reduced local spatial resolution due to heightened kernel sparsity in checkboard patterns. The authors explained their model outcomes using the CAM (Fig. 7).

CAM offers clinicians clear visual explanations by outlining key regions in images, which aids in understanding model decisions. It integrates smoothly with certain neural network architectures that use global average pooling layers. However, its uses are restricted to these specific architectures, reducing its adaptability across different types of models. Furthermore, CAM may fail to identify all critical areas in the image, potentially causing important diagnostic details to be missed.

### 4.5 Grad-CAM for medical images

Nafisah et al. [87] employed different CNNs to compare their performance capabilities across three publicly accessible chests CXR datasets to detect Tuberculosis. The model used sophisticated segmentation networks to extract the region of interest from the X-ray and provide explanations through Grad-CAM. Further, the researchers of [88] showcased a comprehensive framework that combines lesion segmentation and COVID-19 diagnosis from CT scans, focused on utilising an explainable multi-instance multi-task network (EMTN) with Grad-CAM being applied by the authors for analytical purposes. Similar to the preceding study, the VGG-16 was implemented by the [67] for COVID-19 identification and reviewed their model predictions through CAM to foster trust in the intricate architecture. Following this, Ali et al. [66] employed the densely connected squeeze CNNs to classify COVID-19 across four datasets with Grad-CAM being used to evaluate the proposed method. Furthermore, the authors of [65] showcased explanations generated by Grad-CAM, comparing the CNNS against the human benchmark for CT images of COVID-19. Moreover, the Grad-CAM was used to explain the functional structure of brain tumour segmentation models and to derive a visual representation of the internal mechanism that enables networks to perform precise tumour segmentation [24]. Liao et al. [89], introduced a clinically interpretable ConvNet architecture designed for precise Glaucoma detection integrating with Grad-CAM, offering clearer insights by emphasising the specific regions identified by the model. Following this, diverse deep learning architectures, such as the vision transformer, were utilised to train on the Kvasir-capsule image dataset to identify gastrointestinal features from endoscopy images. The authors then employed Grad-CAM for performance comparison and analysis through a heatmap generated by Grad-CAM [69]. A framework designed by [60] for modality classification of medical images, aimed at efficiently organising large datasets with minimal human intervention. The authors highlighted that simpler pre-trained models often outperform complex architectures, especially when dealing with limited datasets. They validated the proposed approach through comparative analysis on the ADNI dataset utilising the Grad-CAM. In addition, the authors of [59] developed an explainable framework for Brain tumour detection, covering segmentation, classification and explanation phases. This framework integrates two streamlined and effective CNNs to thoroughly examine MRI images of Brain tumours and explain the model outcomes using Grad-CAM. Additionally, a lightweight CNN integrated with the Grad-CAM method was utilised for brain tumour detection and localization using the MRI images in [90]. Further, in [71], Inceptionv3 and ResNet50 were implemented to accurately detect chronic lung cancer in CT images. The researchers utilised Grad-CAM to generate the heatmap and provide insights into the decision-making process of the employed architectures.

Grad-CAM is versatile and can integrate with a wide array of CNN architectures, not just those equipped with global average pooling. It generates high-resolution visualisation, improving the localization of important features in medical images. However, the heatmaps produced by Grad-CAM can sometimes be imprecise, failing to clearly pinpoint critical regions, especially in highly detailed or small-scale features within the image. Furthermore, the effectiveness of Grad-CAM largely depends on the specific convolution layer chosen for extracting gradients, which requires fine-tuning to achieve optimal results. Additionally, the researchers evaluated the performance of their CNN architecture but did not mention or utilise any evaluation criteria for the Grad-CAM explanations.

### 4.6 G-Grad-CAM for medical images

The VGG-16 architecture was applied by [67] for identifying COVID-19 and validated their model outcomes through G-Grad-CAM to generate a heatmap to foster trust in the intricate architecture.

G-Grad-CAM combines the Grad-CAM with guided backpropagation to generate high-resolution visualisation that emphasises critical regions affecting model predictions in medical imaging. However, G-Grad-CAM is complex and requires significant computational resources due to its integration of two approaches. Additionally, the guided backpropagation can introduce noise into the visualisations, which complicates the clarity of the interpreted results.

### 4.7 Grad-CAM++ for medical images

Varam et al. [69] utilised various DL-architectures including vision transformer and trained them using the Kvasir-capsule dataset for Gastrointestinal feature identification in endoscopy images. The authors applied Grad-CAM++ for the assessment and comparison of model efficacy, employing its heatmap to visualise findings.

In medical image processing, Grad-CAM++ enhances the Grad-CAM method by providing improved localization capabilities, specifically by addressing the challenges of detecting multiple critical areas within an image. It does this by employing an advanced approach involving weighted combinations of activation maps and the inclusion of higher-order derivatives, allowing for fine detection of small, yet critical features that are vital for accurate medical diagnosis. While Grad-CAM++ generates more refined visual heatmaps, it can produce ambiguous interpretations in cases where significant regions overlap or are closely located, which could challenge the clarity needed in medical diagnostics.

### 4.8 Saliency map for medical image

Stanley et al. [92] implemented and optimized the CNN model for sex classification and demographic subgroup performance analysis. They used the saliency maps to identify important brain regions and investigate it how these regions vary by demographic and their relationship to sex and puberty-associated morphological differences. Furthermore, the CNN architecture integrated with a saliency map was developed by [93] for the automated identification of paediatric papilledema based on optic disc localization and detection of explainable papilledema indicators through data augmentation.

The Saliency map outlining the regions with the highest gradients indicates where slight changes to pixel value can significantly alter the model's predictions, making them useful for understanding model behaviour in diagnostic tasks. On the downside, saliency maps often generate noisy and difficult visualisations, sometimes necessitating additional processing or expert explanation to become useful in a clinical context.

### 4.9 LRP for medical images

Ma et al. [94] conducted a study, that emphasised the use of XAI methods to support the development of trustworthy AI models in dentistry. The authors used LRP to provide a practical demonstration of caries prediction on near-infrared light-transillumination images. Additionally, the Generative Adversarial Networks (GANs) with the implementation of LRP were presented by [61] for Pneumonia detection in the X-ray images. Furthermore, a clinical decision support engine was presented by [91] that leverages MRI images for diagnosing Temporomandibular Joint Disorder (TMJ-ADD) utilising two DNN models. The authors employed LRP to generate a heatmap as a visual explanation for its diagnostic predictions. Following this, another DL-based system was introduced by [95], to detect the Brain Tumour in the multiparametric MRI, T1-weighted and diffusion-weighted imaging and validated the system for an independent cohort of emergency patients. The authors applied LRP for generating heatmap, showing a high overlap of relevance in solid portions of tumours, but not in non-tumours regions. In addition, the LRP was utilised by [86], to explain individual classification decisions for patients with Alzheimer's disease based on CNN using MRI images.

**Fig. 8:**
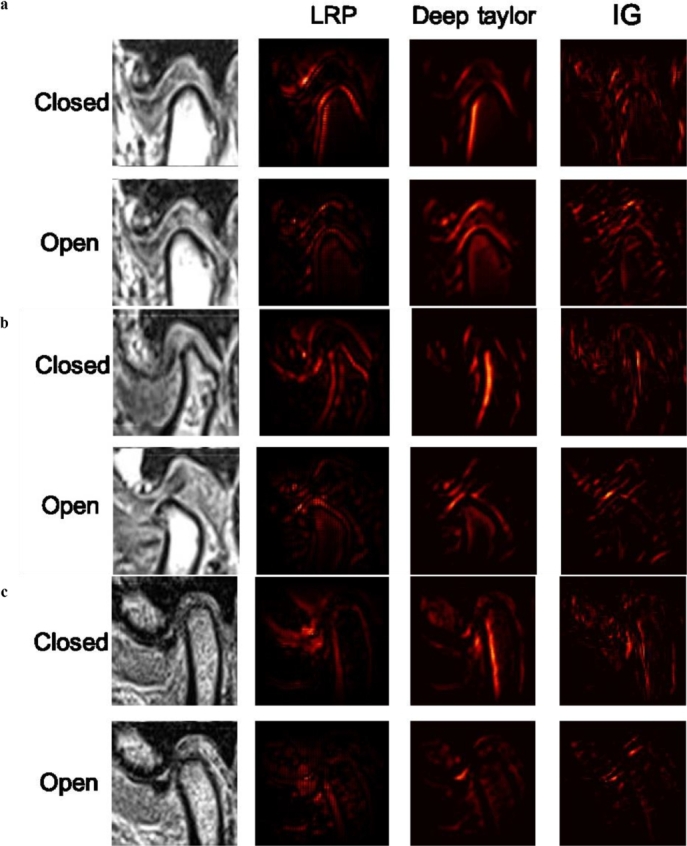
LRP, DTD and IG explanations by [91], present samples of heat maps for three classes: a. normal class, b. ADcR class, and c. ADsR class. The attributions were visualized with heat maps, highlighting important features for each diagnostic case. In all diagnostic cases, the boundary between the three TMJ components in contact with each other was highly activated. In some images, both the surface and the boundary of each component were activated. Despite the different approaches used for calculating explainability, the emphasis was consistently placed on the three TMJ components relevant to the diagnosis of TMJ ADD.

LRP traces the output of neural networks back to the input layer, assigning importance to individual pixels within medical images, effectively highlighting key features in MRI and CT scans. However, the effectiveness of LRP depends heavily on the architecture of the neural network used, which restricts its applicability to certain types of medical imaging. Additionally, LRP occasionally overemphasises regions that lack clinical relevance, which mislead healthcare professionals (Fig. 8).

### 4.10 Surrogate model for medical images

Singla et al. [96] employed the DenseNet-121 architecture, training it on X-ray images and utilising a surrogate model to elucidate the model process. This study sought to offer explanations mirroring the decision-making approach of domain experts articulated in terms of clinicians fine understandable. Surrogate models in medical image processing, are employed to approximate the behaviour of more complex architecture enabling faster analysis and more efficient interpretation. It's useful for rapid testing and analysis allowing clinicians to explore different diagnostic scenarios with less computational overhead. However, one significant limitation of surrogate models is that they do not achieve the same accuracy as more complex models, as they do not capture all nuances of the data leading to oversimplified or incorrect interpretations.

### 4.11 IG for medical images

A clinical decision support engine was presented by [91] that leverages MRI images for diagnosing Temporomandibular Joint Disorder (TMJ-ADD) utilising two DNN models. The authors employed IG to provide a visual explanation for its diagnostic predictions. IG offers a more detailed and theoretically grounded explanation of model decisions, which is particularly useful for identifying influential regions in medical images. However, its effectiveness depends heavily on the baseline selection, which can greatly influence the attributions and lead to potentially inaccurate explanations if poorly chosen. Additionally, the approach can be computationally intensive for high-resolution images as it requires multiple gradient computations along the input path. These drawbacks limit its practicality in a real-time clinical setting.

### 4.12 Counterfactual explanations for medical images

The Blackbox counterfactual explainer method was proposed by [82], to clarify medical image classification overcoming the limitations of traditional interpretability tools. The authors utilised GAN trained and tested on an X-ray dataset to produce the counterfactual images that illustrate the impact of modifying specific features on classification results. Bhattacharya et al. [76] designed an explanation dashboard that predicts the risk of diabetes onset and employed the counterfactual method to explain the important features of the model's decision. In addition, the DenseNet-121 model was trained on X-ray images and integrated with the counterfactual explanations method for pinpointing the architecture outcomes procedures [96]. The study aimed to provide insights aligned with the decision-making patterns of domain experts, presented in terms easily graspable by clinicians. In continuation, the GANs with the implementation of counterfactual explanations were presented by [61] for Pneumonia detection in the X-ray images. In medical image analysis, counterfactual explanations help the clinician understand how altering specific input features changes a model's decision, thereby providing actionable insights crucial for personalized medicine. However, generating clinically relevant and realistic counterfactuals is challenging, as it demands a deep understanding of the model context to ensure the suggested modifications are meaningful and practical. Moreover, creating these explanations requires significant executions particularly when pinpointing minimal changes needed for different diagnosis, which not be feasible in urgent care setting.

### 4.13 OA for medical images

Goel et al. [65] employed the CNN-based architecture for the diagnosis of common Pneumonia using CT and X-ray images and interpreted the model's decision utilising the OA. This method involved systematically obscuring different portions of the image to identify which areas most influence CNN's predictions, providing deeper insight into how the model discerns features indicative of Pneumonia. In addition, the VGG-16 architecture was applied by [67] for identifying COVID-19 and validated their model outcomes through OA to foster trust in the intricate architecture.

OA is computationally slow and needs several forward passes through the model for each version of the image with occluded sections. Furthermore, this approach did not provide precise localisation of relevant features, as the occlusion of larger regions can lead to ambiguous or generalised interpretations of feature importance.

### 4.14 RISE for medical images

This approach is only used by [65], where they applied the CNN architecture to diagnose common Pneumonia from CT and X-ray images and explained the output utilizing the RISE. This method involved systematically obscuring different portions of the image to identify which areas most influence CNN's predictions, providing deeper insight into how the model discerns features indicative of Pneumonia.

RISE does not rely on model gradients, making it applicable across different types of models. It excels by generating pixel-level importance scores, providing detailed insights essential for medical diagnosis. However, RISE requires multiple iterations with different masked inputs to ensure accurate results. Additionally, the randomness in mask application can sometimes lead to variability in the importance scores, which require averaging over multiple executions to stabilise the explanation.

### 4.15 PI for medical images

Khater et al. [97] used the XG-Boost method to understand the lifestyle factors that influence weight levels and identify critical features for weight classification. PI and partial dependence plots were implemented to interpret the proposed model results.

In medical image analysis, PI highlights which image pixels are most critical for accurate diagnosis. However, PI may not provide reliable results in cases with highly correlated features, as shuffling one feature could inadvertently affect the interpretation of another.

### 4.16 MSA for medical images

The ensemble ML models were trained on three different datasets to detect cybersickness and chronic pain by [77]. The authors utilised MSA to explain the model output and identify the dominant features.

MSA provides a global sensitivity measure which is beneficial for understanding complex interactions between multiple variables in medical imaging models. However, it is less accurate when dealing with highly nonlinear interactions, as it oversimplifies the effects of inputs on the output.

### 4.17 GAR for medical images

Mondal et al. [98] reported the use of vision transformers instead of CNN for COVID-19 utilising X-ray and CT scans. They applied multi-stage transfer learning approaches to address data scarcity and explained the features learned by the transformer using the GAR methods.

In medical image processing, GAR provides layer-specific insights and is effective for visualising and understanding the regions in medical images that are most influential for model predictions. On the other hand, it is sensitive to the specific architecture and initialisation of neural networks, potentially leading to variability in the explanations it generates. Additionally, this approach can be affected by noise in the calculations, which hides how important some inputs are.

### 4.18 Attention-based model for medical images

Attention-based model, EXAM is introduced by [99] for COVID-19 automatic diagnosis. EXAM incorporates channel-wise and spatial-wise attention mechanisms to improve feature extraction and explainability.

The attention-based model provides a more focused analysis and misses smaller, yet important details that are less obvious and crucial for a complete diagnosis.

### 4.19 AS for medical images

Olar et al. [100] created a best-performing and explainable model that maps clinical metadata to image features to predict the COVID-19 prognosis. The researchers applied various ML methods for correctly diagnosing the severity of COVID-19 based on X-ray images collected upon admission to the hospital along with clinical metadata. Afterwards, the AS were conducted to identify the crucial parts of the models and the predictive power of each feature in the dataset. Similar to the preceding work, the VGG-16 was implemented by [67] for COVID-19 identification and reviewed their model predictions through AS to foster trust in the intricate architecture.

In medical image analysis, AS are particularly useful for identifying redundancies and inefficiencies in a complex architecture, aiding in the refinement and simplification of the system without sacrificing performance. However, the explanation of AS results can be challenging, as removing one component might inadvertently affect others and it's complicating the understanding of each part's true impact.

### 4.20 DTD for medical images

A clinical decision support engine was presented by [91] that leverages MRI images for diagnosing Temporomandibular Joint Disorder (TMJ-ADD) utilising two DNN models. The authors employed DTD to provide explanation for its diagnostic predictions.

DTD is grounded in Tylor series expansion, offering a mathematically rigorous approach that enhances the transparency of complex models. However, its accuracy depends on the selection of the root point for the Taylor expansion, which introduces subjectivity and variability in the explanations. Moreover, while effective for architectures with ReLU activation functions, its implementation is less straightforward in architecture using different types of nonlinearities.

Table 3 summaries various XAI methods used in medical imaging. We organised these methods by modality, including MRI, CT, Fundus, Endoscopy and X-ray, and linked them to specific diseases.

**Table 3:** Application of XAI methods in medical imaging.

XAI Approach	Modality	Disease	References
LIME	MRI	Alzheimer, Thyroid	[70], [68]
	CT	Lung cancer, Covid-19	[101], [71]
	X-ray	Covid-19, Pneumonia	[101], [67], [66], [65], [64]
	Fundus Image	Retinoblastoma, Glaucoma	[62], [79]

SHAP	MRI	Alzheimer, Breast cancer	[70], [81]
	CT	Renal cell carcinoma, Kidney injury	[79], [80]
	X-ray	Covid-19, Coronary Artery Disease	[73], [75], [72]
	Fundus Image	Retinoblastoma	[58]
	Endoscopy	Gastrointestinal	[69]

CAM	MRI	Brain tumour, Knee injury, Knee pain	[83], [84], [85]
	CT	Brain tumour	[59]
	X-ray	Breast cancer, Osteoarthritis severity	[82], [102]

Grad-CAM	MRI	Brain cancer, Alzheimer, Glaucoma	[90], [59], [60], [63], [24], [89]
	CT	Lung cancer, Covid-19	[71], [88], [65]
	X-ray	Tuberculosis, Covid-19	[65], [87], [67], [66]
	Fundus Image	Gastrointestinal	[69]

G-Grad-CAM	X-ray	Covid-19	[67]
Grad-CAM++	Fundus Image	Gastrointestinal	[69]

Saliency Map	MRI	Black and White adolescents	[92]
	Fundus Image	Papilledema	[93]

LRP	X-ray	Dentistry, Pneumonia	[94], [61]
	MRI	Temporomandibular joint anterior disk displacement, Brain tumour, Alzheimer	[91], [95], [86]

Surrogate Model	X-ray	Chest diseases	[96]

IG	MRI	Temporomandibular joint anterior disk displacement	[91]

Counterfactual explanations	X-ray	Chest diseases, Enlarged cardio-mediastinum cardiomegaly, lung-lesion, Lung-opacity, Edema, Consolidation, Pneumonia, Atelectasis, Pneumothorax, Pleural effusion, Pleural other, fractures	[96], [103], [76]

OA	X-ray	Covid-19	[65], [67]
	CT	Covid-19	[65]

RISE	X-ray	Covid-19	[65]
	CT	Covid-19	[65]

PI	Multi-modality	Obesity	[97]

MSA	Multi-modality	Cybersickness	[77]

GAR	X-ray	Covid-19	[98]
	CT	Covid-19	[98]

Attention-based	X-ray	Covid-19	[99]
	CT	Covid-19	[99]

AS	X-ray	Covid-19	[67]

DTD	MRI	Temporomandibular joint anterior disk displacement	[91]

## 5 Discussion/limitations and future directions

In this paper, we delve into the application of XAI methods specifically within the context of medical imaging data. While these approaches demonstrate promising outcomes, they show good to excellent but integrating them into clinical practices poses several challenges. Our systematic literature review reveals key obstacles and considerations essential for their successful integration into healthcare. Our findings offer a clear direction for future research, proposing the possibility of more transparent, understandable and patient-focused AI applications in the medical field.

### 5.1 Limitation of attribution maps in medical practice

In XAI, particularly within medical image analysis, saliency-map-based methods have emerged as crucial tools for enhancing the model explanations. However, these approaches are accompanied by technical limitations that can impact their effectiveness and reliability. Although numerous extant methods based on saliency maps highlight the important pixel of an image, they have often fallen short in various evaluation tests. Gradient * Input (GI) [104], a technique in XAI that multiplies the gradient of the model's output with respect to the input by the original input itself, enhances the sharpness of heatmaps, providing clearer and more interpretable visualizations of important features. Furter, Guided Backpropagation (GB) [105], another technique in Explainable AI, enhances model interpretability by visualizing important input features. It modifies standard backpropagation to only allow positive gradients, creating clearer and more focused attribution maps. For instance, four attribution methods such as GI, GB, LRP and OA were tested against robustness for the classification of Alzheimer's disease but failed against the robustness in several experiments [106]. Furthermore, the extant literature shows that the attribution method fails in randomization evaluations. As an example, Adebayo et al. [107] demonstrated that approaches like GB and G-Grad-CAM could produce a visual explanation without proper training. As such, the application attribution map-based approaches to medical imaging need careful evaluation, emphasising the need for future studies to enhance the robustness, effectiveness, reproducibility and consistency of attribution systems in generating saliency maps.

### 5.2 Limitation of non-attribution approaches

The existing non-attribution methods such as counterfactual and concept-based learning face challenges including computational intensity, the requirement for domain-specific knowledge or significant annotation expanses. For instance, a primary hurdle with concept learning approaches is their dependency on the manual selection of concept examples by humans leading to increased annotation costs. Some other drawbacks are that a misleading explanation is possible due to confounding concepts and concepts may not causally affect the model's decision [107]. On the other hand, one of the limitations of counterfactual explanations is their reliance on image perturbation techniques that may produce unrealistic outcomes. Additionally, the generation of counterfactual images requires an autoencoder, meaning that the representation can suffer from low-quality or insufficient data. Thus, enhancing the image perturbation process should be a priority.

### 5.3 Insufficient evaluation metrics

Despite the advancements in applying XAI methods to medical image analysis, a significant gap remains in evaluating these methods. No authors used evaluation metrics specifically for explainability in the papers considered in this review. Currently, the evaluation metrics in use are those already established within DL and computer vision, which may not adequately address the nuances of explainability in AI. This gap highlights a significant opportunity for future research to develop specialised evaluation methods tailored to XAI. The development of such quantitative evaluation metrics faces a major obstacle due to the intangible nature of establishing a definitive ground truth for explanations. Consequently, this area represents a promising avenue for future research, considering the field's relative infancy and the critical need for more precise and applicable evaluative criteria.

### 5.4 Trade-off between interpretability and accuracy

In DL, a prevalent misconception exists that a trade-off between interpretability and accuracy; that is, a model's high accuracy often comes at the expense of its explainability and conversely, more explainable models typically demonstrate lower accuracy. However, emerging evidence suggests this trade-off might not hold true; improved interpretability could lead to enhanced accuracy [108]. This insight opens new avenues for future research to implement and develop new XAI methods that have high explainability as well as outstanding performance.

### 5.5 Complex architecture

Another future research direction could focus on investigating the performance of XAI approaches within complex architectures. Existing evaluations of XAI methods predominantly focus on shallow models where tools like the influence function can provide precise outcomes. However, the transition to deeper and more complex architecture introduces a challenge, as these methods tend to yield inaccurate results when dealing with increased complexity [109]. Therefore, the mentioned observation raises important questions about the adaptability and reliability of current XAI techniques when confronted with complex DL models. Hence, it highlights the need to improve the existing XAI methods for complex architectures and ensure that explainability keeps pace with the growing complexity of the models.

### 5.6 Multimodal data

Exploring XAI techniques within the context of multimodal datasets reveals another dimension of potential future research. To date, XAI methods have been utilised for simple datasets, while medical image datasets are often complex patterns and comprehensive attributes, offering a distinct challenge to existing frameworks. Multimodal medical datasets, in particular, cover a wide range of data types such as X-ray, MRI, CT, microscopic, etc, demanding a more sophisticated approach to both explanation and interpretation. Thus, there is an essential need for the XAI research community to extend its focus towards developing and testing explainability methods that are robust and effective across the diverse landscapes of the multimodal dataset.

### 5.7 Computational cost

The computational costs of XAI techniques vary significantly and present notable limitations and challenges. Perturbation-based methods, such as LIME and SHAP, are particularly computationally expensive due to the need for numerous model evaluations and the retraining of surrogate models, resulting in high overhead [110]. This makes them less feasible for real-time medical imaging applications. In contrast, gradient-based methods (Grad-CAM, G-Grad-CAM, Grad-CAM++, Saliency map, LRP, RISE, GAR, Attention-based and DTD) are generally more efficient. For instance, Grad-CAM requires only a single backpropagation pass, making it suitable for real-time or near-real-time applications in medical imaging [111]. Similarly, methods like IG and DTD leverage gradient information efficiently, offering detailed and timely explanations. Activation-based methods, such as CAM, are relatively efficient as they utilize pre-computed activation maps during the forward pass. CAM, for example, involves a straightforward weighted sum of activation maps, making it computationally efficient [36]. Despite these efficiencies, careful consideration of computational costs is essential when choosing the appropriate XAI method for medical imaging applications.

## 6 Conclusion

In this systematic literature review, we explored the latest developments in XAI employed in medical image analysis. Furthermore, we detailed 18 distinct XAI approaches providing clear explanations of their definitions, foundational concepts and the mathematical framework applied within the context of medical imaging. Additionally, this study systematically presents the current challenges and limitations of each method, thereby assisting researchers in meticulously selecting the appropriate XAI method tailored to their specific problem.

To strengthen the future of XAI in medical imaging, it is crucial to focus on developing more robust evaluation metrics, enhancing the integration of XAI with clinical workflows, and designing complex architectures that inherently support explainability without compromising accuracy. Additionally, integrating XAI with multimodal data and combining multiple AI models (ensemble and hybrid) for comprehensive evaluation will further enhance the reliability and applicability of XAI methods in clinical settings.

## CRediT authorship contribution statement

**Dost Muhammad:** Writing – review & editing, Writing – original draft, Visualization, Validation, Resources, Methodology, Investigation, Funding acquisition, Formal analysis, Data curation, Conceptualization. **Malika Bendechache:** Writing – review & editing, Visualization, Validation, Supervision, Project administration, Methodology, Investigation, Funding acquisition.

## Declaration of Competing Interest

The authors have no conflict of interest.

## Footnotes

1 https://www.fda.gov/media/109618.

2 Data was only collected until first quarter of 2023.
